# Pseudorange Measurement Method Based on AIS Signals

**DOI:** 10.3390/s17051183

**Published:** 2017-05-22

**Authors:** Jingbo Zhang, Shufang Zhang, Jinpeng Wang

**Affiliations:** 1 Information Science and Technology College, Dalian Maritime University, Dalian 116026, China; sfzhang@dlmu.edu.cn; 2 School of Information Science and Engineering, Dalian Polytechnic University, Dalian 116034, China; wangjp@dlpu.edu.cn

**Keywords:** pseudorange measurement, Gaussian baseband, timestamp detection, optimal estimation, AIS-R mode

## Abstract

In order to use the existing automatic identification system (AIS) to provide additional navigation and positioning services, a complete pseudorange measurements solution is presented in this paper. Through the mathematical analysis of the AIS signal, the bit-0-phases in the digital sequences were determined as the timestamps. Monte Carlo simulation was carried out to compare the accuracy of the zero-crossing and differential peak, which are two timestamp detection methods in the additive white Gaussian noise (AWGN) channel. Considering the low-speed and low-dynamic motion characteristics of ships, an optimal estimation method based on the minimum mean square error is proposed to improve detection accuracy. Furthermore, the α difference filter algorithm was used to achieve the fusion of the optimal estimation results of the two detection methods. The results show that the algorithm can greatly improve the accuracy of pseudorange estimation under low signal-to-noise ratio (SNR) conditions. In order to verify the effectiveness of the scheme, prototypes containing the measurement scheme were developed and field tests in Xinghai Bay of Dalian (China) were performed. The test results show that the pseudorange measurement accuracy was better than 28 m (σ) without any modification of the existing AIS system.

## 1. Introduction

As ships sailing on the sea lack reference, a dependable technological means of position, navigation, and timing (PNT) is an important guarantee of ship navigation safety. Nowadays, global navigation satellite systems (GNSS) are the main source of PNT information in the maritime domain. Unfortunately, there are certain risks in using only GNSS, which are vulnerable to jamming or interference [1,2,3]. Thus, the International Maritime Organization (IMO) called on the research institutions which are engaged in maritime information technology to study innovative technology in alternative backup navigation systems [4].

In addition to GNSS, shore-based radio navigation systems (SRNS) [5,6], inertial navigation systems (INS) [7,8,9], and celestial navigation systems (CNS) [10,11,12] have been used in the maritime domain. INS and CNS are used mainly in military applications due to cost factors. The Loran-C system (one of the SRNS) is widely used in the civil field [13,14] and was first developed successfully by the United States, with China and Russia developing corresponding systems (Changhe-2 and CHAYKA) [15]. However, due to low user usage, the United States shut down the system in 2010 [16]. Therefore, subsequent shore-based backup systems should meet reliability and economic requirements.

There have been two main development directions in SRNS. The United States has discussed the feasibility of an eLoran system since 2015 [17]. In order to avoid the tragedy of Loran-C, the new generation system plans to provide time service to land users based on ships [18]. The European Union (EU) and China are interested in using the existing AIS shore station to build new backup location systems, with a related technique called automatic identification system range (AIS-R) mode.

AIS is the most widely-used system in the maritime wireless communication field. According to the IMO correlative standards, vessels are mandated to be equipped with automatic identification system (AIS) ship-borne terminals [19], and the IMO’s major member states have to locate a series of AIS base stations along coastlines to realize data exchange between ship and shore. Currently, AIS plays an important role in the field of maritime safety management, therefore the study of AIS-R mode without increasing maintenance and owner costs has a feasible market application value as a backup positioning system.

In the EU, research of AIS-R mode is an important part of the Accessibility for Shipping, Efficiency Advantages and Sustainability (ACCSEAS) project. Current progress includes a completed technical feasibility study [20], but has not yet provided corresponding technical solutions. In China, the research team of Dalian Maritime University has developed prototypes and carried out various field tests [21]. In this paper, the key technology of the pseudorange measurement method is described in detail.

The rest of the paper is organized as follows. Section 2 introduces the basic principle of pseudorange measurement, including the time of arrival (TOA) measurement method and timestamp option. Section 3 discusses the detective feature of timestamp, including amplitude characteristics and differential characteristics, and analyzes the influence of adjacent bits in digital message sequence on the timestamp detection characteristic. Section 4 compares the detection accuracy of zero-crossing detection and differential peak detection under different signal-to-noise ratio (SNR) conditions. Section 5 studies the optimal estimation algorithm based on minimum mean square error and the fusion algorithm based on α difference filtering. In order to validate the proposed measurement scheme, field tests in Xinghai Bay of Dalian (China) are provided in Section 6. Finally, some conclusions are put forward in Section 7.

## 2. Principle of Pseudorange Measurement in AIS-R

### 2.1. The TOA Measurement Method

The range measurement system based on radio signals is essentially a transmission delay measurement system [22]. At the same time benchmark, the transmitter sends out an electromagnetic wave with easily monitored features at the appointed time *t_T_*, then the receiver detects the signal feature and records the reception time *t_R_*. The difference between the two data is the transmission delay Δ*t*. Transmission delay multiplied by the speed of an electromagnetic wave *c* is the distance between the transmitter and receiver *d*.

(1) $$d=c\times \Delta t=c\times \left(t_{R}-t_{T}\right)$$

The method for calculating the distance *d* by calculating the signal transmission delay Δ*t* is known as the time of arrival method. In practical application, as there is no guarantee that the receiver and transmitter adopt the same time benchmark, the result of Equation (1) contains a system clock error, thus distance *d* is called the pseudorange.

### 2.2. Timestamp Option

The special symbols containing emission timing information in wireless transmission signals are called timestamps. Selecting easily monitored timestamps and designing their detection model are the basis of any TOA measurement system. 

Usually in TOA measurement systems, timestamps are in an arbitrary carrier phase or code phase. AIS is typically communications based, which is not primarily intended for positioning so it is impossible to measure the arbitrary code phase. Based on this factor, AIS uses Gaussian minimum shift keying (GMSK) modulation, where the carrier frequency changes in a linear fashion. When the current carrier phase is detected, the carrier frequency cannot be obtained, thus the launching time *t_T_* cannot be determined. A correlation detection method based on a fixed message format is proposed in the literature [23], which wastes the limited AIS communication bandwidth. 

**Figure 1:**
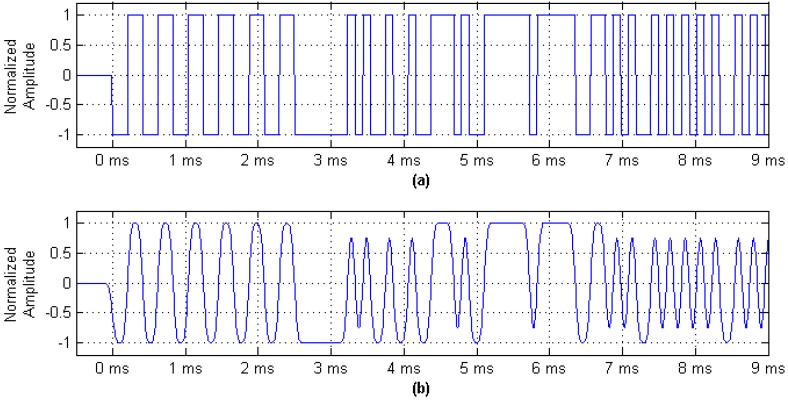
AIS digital message sequence and Gaussian baseband waveform. (**a**) AIS message digital sequence waveform; and (**b**) Gaussian minimum shift keying (GMSK) baseband wave form.

The initial transmission time of each frame signal can be determined as the AIS uses time division multiple address (TDMA) networking mode, so the launch time *t_p_*_0_ of every bit-0-phase in the frame can be calculated. The bit-0-phase corresponds to the hopping edge in the digital message sequence shown in Figure 1a. These features are easily detected by the receiver, so the bit-0-phase that contains launch time is regarded as the timestamps in the paper.

Considering that AIS adopts the GMSK modulation method, the reproductive baseband signal in the receiver smoothly changes the Gaussian baseband waveform shown in Figure 1b, before the receiver obtains the binary bit sequence by the bit decision algorithm, which only focuses on the bit error rate and is not concerned with the launch time information of the bit-0-phase. Therefore, the research subject can only be the Gaussian baseband waveform rather than the digital message sequence. Thus, the issue of how to accurately detect the bit-0-phase in the smooth Gaussian baseband waveform first needs to be resolved.

### 2.3 Measurement Scheme

**Figure 2:**
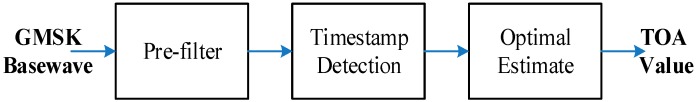
Scheme block diagram.

The specific program for AIS signal transmission delay measurement is shown in Figure 2.

In this scheme, the pre-filter is used to suppress the interference caused by various types of noise in the Gaussian baseband waveform. Taking into account that the ideal baseband waveform should be a Gaussian waveform, the pre-filter uses a Gaussian low-pass filter.

In order to get these timestamps in a smooth Gaussian baseband waveform, we proposed two detection algorithms (zero-crossing detection and differential peak detection) to detect the bit-0-phases in the baseband waveform. As one of the key research contents of this research, the mathematical model of these schemes is analyzed theoretically in Section 3. In Section 4, the detection accuracy of these schemes under AWGN channel is simulated and analyzed.

Since the existing AIS system is a communication system rather than a professional pseudorange measurement system, the detection accuracy of a single timestamp cannot meet the requirements of measurement accuracy. Considering the slow-motion characteristics of the ship, the optimal estimation algorithm is used to improve the estimation accuracy of the detection algorithm. The corresponding contents are discussed in detail in the Section 5.

## 3. Detective Feature of Timestamp

### 3.1. Gaussian Filter System Characteristics

The signal characteristics of the Gaussian baseband waveform are completely determined by the Gaussian filter, which is a low-pass filter in which unit impulse response satisfies the Gaussian distribution where the mean value is 0 [24].

(2) $$h(t)=\frac{1}{\sqrt{2\pi }\delta }\exp\left(-\frac{t^{2}}{2\delta^{2}}\right)$$

where *δ*^2^ is the variance of Gaussian distribution, and *δ* is standard deviation. The probability density function of the Gaussian distribution is bell-shaped, and the parameter *δ* determines the magnitude of the curve distribution. 

In the field of signal processing, the bandwidth-time product *BT* is usually used to characterize the Gaussian filter in which *B* is the 3 dB bandwidth of the filter, and *T* is the bit period. *BT* and *δ* satisfy the following definition

(3) $$\delta =\frac{\sqrt{\ln2}}{2\pi \left(BT\right)}$$

Equation (3) is introduced into Equation (2) to obtain another expression of Gaussian filter unit impulse response

(4) $$h(t)=\frac{\sqrt{2\pi }\left(BT\right)}{\sqrt{\ln2}}\exp\left(-\frac{2\pi^{2}{\left(BT\right)}^{2}}{\ln2}t^{2}\right)$$

For subsequent discussion, the definition of parameter *k* meets the following equation:

(5) $$k=\sqrt{\frac{2}{\ln2}}\pi \left(BT\right)$$

Correspondingly, the unit impulse response function of the Gaussian filter expressed by Equation (4) can be simplified as

(6) $$h(t)=\frac{k}{\sqrt{\pi }}e^{-k^{2}t^{2}}$$

Thus, it can be seen that the baseband waveform characteristics in the GMSK communication system are only relevant for parameter *BT*. Usually the smaller the *BT* value, the faster the attenuation of the GMSK high-frequency component, but at the same time, the greater the interaction between adjacent bits. Therefore, in GMSK communication systems, the parameter *BT* should be considered synthetically.

### 3.2. Amplitude Characteristics of Timestamp

When the bit period *T* tends to be infinite, the output result that a rising edge of the digital message sequence passes through the Gaussian filter is equivalent to the unit step response of the Gaussian filter.

It is well known that in a linear system, the unit step response is the integral of the unit impulse response. In addition, since the unit impulse response function of the Gaussian filter is an even function regardless of the value of the parameter *k*, the following ∞ conclusions can be obtained.

(7) $$\int_{-\infty }^{+\infty }h(x)dx=1$$

Therefore, the unit step response function of the Gaussian filter *a*(*t*) can be expressed as

(8) $$\begin{array}{ll} a(t) & =\int_{-\infty }^{t}h(x)dx=\int_{-\infty }^{0}h(x)dx+\int_{0}^{t}h(x)dx\\ \; & =\frac{1}{2}+\int_{0}^{t}\left(\frac{k}{\sqrt{\pi }}e^{-k^{2}x^{2}}\right)dx=\frac{1}{2}+\frac{1}{\sqrt{\pi }}\int_{0}^{kt}e^{-u^{2}}du\\ \; & =\frac{1}{2}\left[1+erf\left(kt\right)\right] \end{array}$$

**Figure 3:**
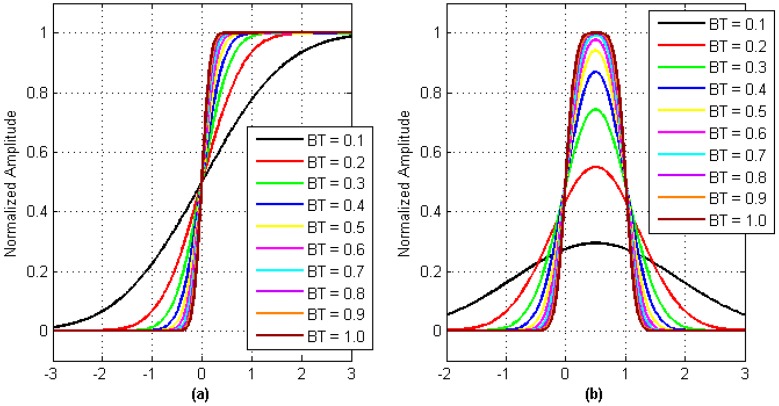
Relation between unit response of Gaussian Filter and parameter *BT*. (**a**) Gaussian filter unit-step response; and (**b**) Gaussian filter unit impulse response.

On the condition that the parameter *k* (defined in Equation (6)) is introduced to Equation (8), we can obtain the relational expression between the unit step response of the Gaussian filter and the parameter *BT*. When parameter *BT* takes different values, we get the corresponding unit step response curve shown in Figure 3a. As seen in the figure, for the Gaussian baseband waveform recovered by the receiver, the median point is the output result of the digital message sequence bit-0-phase that can be treated as the timestamp.

#### 3.2.1. Interference of Adjacent Bits to Timestamp Amplitude Characteristics

When the unit step response of the Gaussian filter is discussed, the bit period *T* is approximated to infinity. In practice, the alternately transformed sequence bits introduce amplitude distortion into the Gaussian baseband waveform. As shown in Figure 3b, the amplitude of the unit pulse signal bit hopping edge in the Gaussian baseband waveform is no longer deterministic.

The digital message sequence can be thought of as the result of a series of shifted unit step signal alternate additions. Since the Gaussian filter is a linear time-invariant system, the output of the Gaussian filter is equivalent to the interleaved summation of a set of translational unit step responses of the Gaussian filter for GMSK modulation systems.

(9) O(t)G=∑m=0N{(−1)m×[1+erf(k(t−mT))]}

where *T* is the bit period in digital message sequence; *m* is the bit serial number of the bit inversion generated in the digital message sequence, and is a monotonically increasing random sequence of positive integers; *N* is the number of bit inversion in the finite-length digital message sequence, and its value is less than or equal to the number of the sequence. 

For the convenience of subsequent discussion without loss of generality, we defined the digital message sequence as a 0 or 1 alternating unit periodic sequence. Under such conditions, in Equation (9), *T* = 1 and m∈{0,1,2,⋯N}, where *N* is the number of bits.

Let b(t)j=erf[k(t−2j)]−erf[k(t−(2j+1))], Equation (9) can be expressed as

(10) O(t)G={∑j=0(N−1)2b(t)jN is odd∑j=0(N2)−1b(t)j+[1+erf(k(t−N))]N is even

As the error function erf(⋅) is monotonically increasing, we obtain the following conclusions: b(t)j>0 and b(t)j<b(t)j+1. According to the D’Alembert discriminant method, it is known that the series ∑j=0∞b(t)j is convergent.

**Figure 4:**
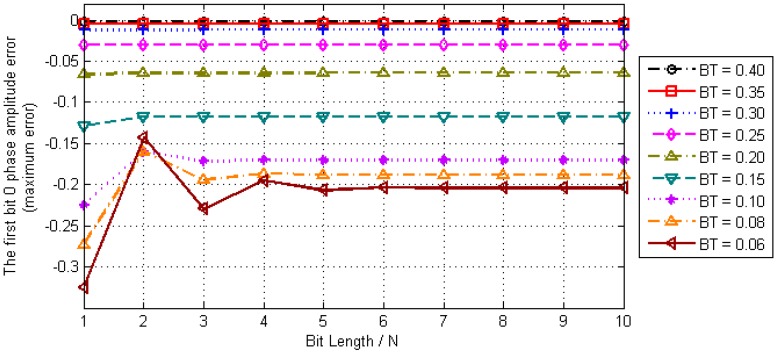
Convergence Analysis of *O_G_*(0).

When *t* is a positive integer, the result *O_G_*(*n*) is the amplitude of bit hopping edges that we want to detect timestamps in the Gaussian baseband waveform. Let *t* = 0 in Equation (10) and perform numerical analysis where the amplitude error of the first timestamp *O_G_*(0) is shown in Figure 4.

It can be seen from the results of numerical analysis that *O_G_*(0) is ultimately convergent with the increase of the length of sequence *N*. However, for different parameters *BT*, the convergence rate and the final convergence value of *O_G_*(0) are also different, where the larger the *BT* value, the smaller the convergence error and the faster the convergence rate.

**Figure 5:**
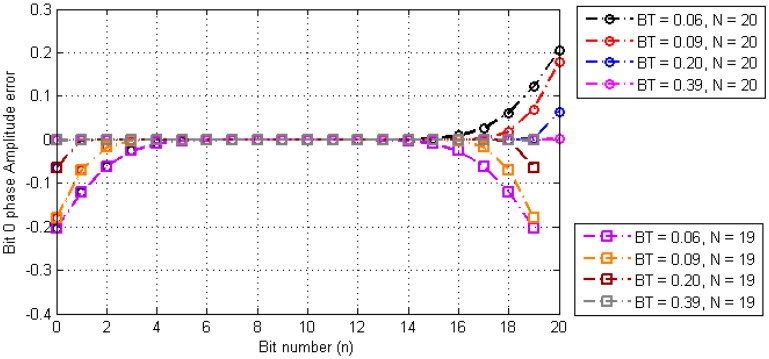
Amplitude error of the timestamps.

Assuming that sequence length *N* is long enough to converge the series expression, the amplitude error of each timestamp of the digital sequence in the Gaussian baseband waveform is obtained by numerical analysis. The corresponding results are shown in Figure 5.

From the numerical analysis, results can be drawn as follows:

- The amplitude error of the timestamp is the largest at both ends of the digital message sequence, and is the smallest in the midpoint of the sequence. The error gradually converges from the two ends to the middle.
- When *N* is an odd number, the amplitude error of the timestamp in the digital messages sequence is in even symmetry with respect to the midpoint of the sequence.
- When *N* is an even number, the amplitude error of the timestamp in the digital messages sequence is in odd symmetry with respect to the midpoint of the sequence.
- When *BT* is smaller, the bigger the error, the slower the convergence.

According to the above conclusions, it can be determined that the amplitude characteristic error introduced by adjacent bit disturbances in the Gaussian baseband waveform can be neglected if the appropriate timestamps are selected.

#### 3.2.2. Timestamp Selection Scheme in AIS-R Mode

Based on the conclusions of the previous section, it can be seen that the errors between the outputs are obtained by single pulse signal hopping edges through the Gaussian filter and the amplitude midpoint obtained by the unit step signal hopping edge through the Gaussian filter is greatest.

For a single pulse signal, Equation (9) can be simplified as

(11) O(t)G={erf(kt)−erf[k(t−1)]}=2π∫k(t−1)kte−η2dη

As the integrand function $e^{-\eta^{2}}$ is even function, so 

(12) O(0)G=O(1)G=12erf(2πBTln2)

For AIS systems, the maximum normalized amplitude error of the bit-0-phase in the Gaussian baseband waveform is about 1.27 × 10^−3^ due to *BT* = 0.4 [25].

In the AIS system, the minimum length of each frame packet is 56 bits [25]. The conditions *N* = 56 are brought into Equation (10) for numerical analysis. The results are shown in Table 1.

From the results of the numerical analysis shown in Table 1, it can be seen that in addition to each two bits at the beginning and end of an AIS packet, the remaining bit-0-phases can be used as the timestamp for measuring the transmission delay in the AIS-R mode.

#### 3.2.3. Timestamp Decision Threshold

According to the previous analysis, the amplitude midpoint of the Gaussian baseband waveform is the bit-0-phase of the digital message sequence. Therefore, the decision threshold depends on the magnitude of the baseband signal, which is related to signal strength. In general, this means that the decision threshold is not a fixed value and increases the complexity of the detection as well as introducing additional detection errors.

However, in the AIS system, the digital message sequence uses bipolar signal coding, the corresponding unit step signal is expressed as

(13) i(t)=2U(t)−1

where *U*(*t*) is standard unit step signal. 

Since the Gaussian filter is a linear system which has homogeneity and superposition, the output result corresponding to the input signal *i*(*t*) is

(14) $$\begin{array}{ll} O(t) & =2a_{h}(t)-1\\ \; & =2\times \frac{1}{2}\left[1+erf\left(kt\right)\right]-1=erf\left(kt\right) \end{array}$$

Ideally, the amplitude decision threshold of the detected timestamp is a fixed value of zero. In this paper, the timestamp detection method based on the amplitude characteristics of the Gaussian baseband waveform is called zero-crossing detection.

### 3.3. Differential Characteristics of Timestamps

The derivative of the ideal Gaussian baseband waveform rising edge function *a*(*t*) (which is defined by the error function) is in accord with the characteristics of the Gaussian function.

(15) $$f(t)=\frac{d}{dt}a(t)=\frac{d}{dt}erf(kt)=\frac{k}{\sqrt{\pi }}e^{-k^{2}t^{2}}=h(t)$$

where the peak point of the differential results corresponds to the bit-0-phase of the digital signal.

To discuss the influence of adjacent symbols on the bit-0-phase differential characteristic, the result of the differential operation on the digital message sequence (defined by Equation (9)) is shown below.

(16) fG(t)=ddtO(t)G=∑m=0N[(−1)m×h(t−mT)]

**Figure 6:**
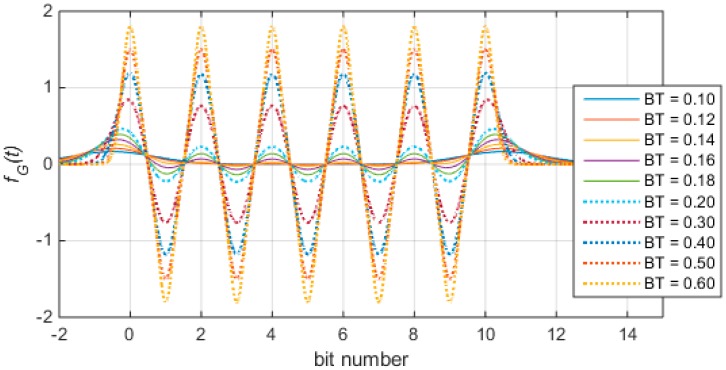
Influence of inter-symbol interference on timestamp differential property.

According to the characteristics of Gaussian function, it is known that the inter-symbol interference is more significant when the interval of the symbol sequence is smaller. Therefore, we still use the unit periodic sequence in which bit 0 and bit 1 alternate as the object of analysis. In Equation (16), let *T* = 1 and m∈{0,1,2,⋯N}, where *N* is the number of bits. Under such conditions, the influence of inter-symbol interference on the timestamp differential property when the parameter *BT* takes different values is shown in Figure 6.

It can be seen from Figure 6 that, when *BT* is greater than 0.2, the influence of inter-symbol interference on the timestamp differential property can be ignored, and when *BT* is greater, the differential peak of bit-0-phase is sharper.

## 4. Accuracy Analysis of Single Timestamp Detection

Based on the results of the previous analysis, we present two methods to detect the timestamp of the Gaussian baseband wave in the AIS system, which are the zero-crossing detection method and the differential peak detection method. Due to the interference of the channel and the thermal noise of the receiver, there is noise interference in the GMSK Gaussian baseband waveform, which is recovered by the receiver demodulator. By considering that the wireless channel usually obeys the AWGN channel model and the thermal noise of the system is also in accordance with the Gaussian characteristic [26], the noise superimposed on the baseband waveform is also consistent with the characteristics of white Gaussian noise. Therefore, it is necessary to filter out the noises from the reproduced Gaussian baseband waveform before performing the timestamp detection.

**Figure 7:**
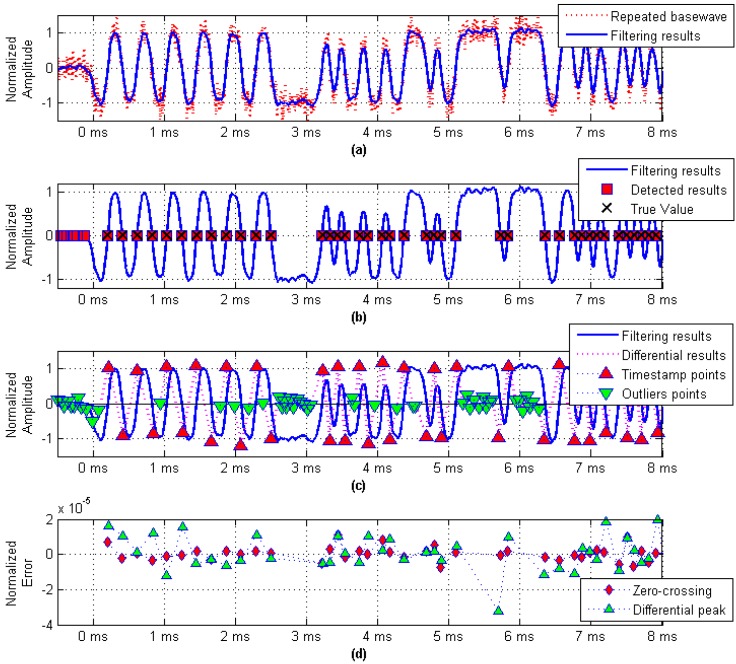
Timestamp detection method. (**a**) Repeated GMSK baseband waveform; (**b**) Zero-crossing detection method; (**c**) Differential peak detection method; and (**d**) Detection error distribution.

As shown in Figure 7a, the SNR is equal to 10 dB, and the noise suppression was performed using a Gaussian filter whose parameter *BT* was set to 0.6. Figure 7b shows the relationship between the real value and the timestamp detection result, which was obtained using the zero-crossing detection method. The results obtained by the differential peak detection method for the same baseband signal are shown in Figure 7c. Since the differential detection method is more sensitive to signal jitter, there will be a large number of outliers. In addition, the amplitude of outliers related to the SNR makes the differential peak detection method much more complex than the zero-crossing detection method in actual use. Figure 7d compares the zero-crossing detection method and the differential peak detection method. It can be seen that the error jitter range of the zero-crossing detection method is smaller than that of the differential peak detection method.

The detection accuracy of timestamps is affected by the SNR of the recovered baseband waveform, so we analyzed the detection accuracy under different SNR conditions.

The simulation parameters were set as follows: (1) the parameter *BT* of the pre-Gaussian lowpass filter was set to 0.6; (2) the length of one message frame was 256 bits; (3) the digital message sequence was a binary random sequence that was different each time; (4) the same Gaussian baseband signal was processed by the zero-crossing detection method and the differential peak detection method to detect timestamp; (5) the process was repeated 20,000 times at a certain signal-to-noise ratio, and the mean and standard deviation of all timestamps detection error were calculated.

All communications systems have the noise floor caused by ambient atmospheric. The equation is as follows

(17) N=kTB

where *k* = 1.38 × 10^−23^ J/K is Boltzman’s constant; *T* is system temperature, usually assumed to be 290 K; *B* is channel bandwidth measured in Hz. This is the lowest possible noise level for a system with a given physical temperature. 

Usually the noise received by the receiver contains additional noise from other sources in addition to the noise floor. A common model for the average additional noise power from other sources is

(18) $$F_{am}=c-27.7\times \log_{10}f$$

in which *f* is the frequency in MHz, *c* is a constant depending upon the channel scenarios. For coastal communication environments, *c* = 72.5 dB [23].

**Figure 8:**
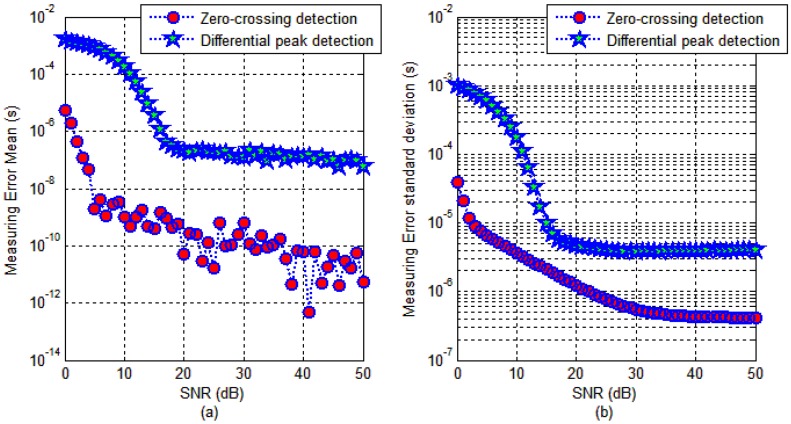
Comparison of timestamp detection results under different SNR. (**a**) Error mean; and (**b**) Error standard deviation.

For AIS receivers, the channel bandwidth *B* = 25 kHz and the carrier frequency *f* = 162 MHz. Using the Equations (17) and (18), we can determine that the AIS receiver noise is −117 dBm. Therefore, when the AIS signal strength is −107 dBm which is the sensitivity required by the AIS standard [23], the corresponding baseband signal SNR is about 10 dB. A typical strong signal is −75 dBm, and the corresponding baseband signal SNR is approximately 42 dB. Thus, the parameter SNR range was set from 0–50 dB in the simulation scheme. The simulation results are shown in Figure 8.

It can be seen in Figure 8 that the zero-crossing detection method is better than the differential peak detection method under the same conditions. The mean of the measurement error is defined as *μ*, and the standard deviation is defined as *σ*. Assuming that the measurement results are normal distribution, according to statistics, we can get that the probability of detection is 68.4% in the range of [*μ* − *σ*, *μ* + *σ*].

When the SNR is greater than 10 dB, the average value of the transmission delay measurement error of the zero-crossing detection method is less than 1.8 × 10^−9^ s, which is equivalent to the pseudorange measurement error better than 54 cm; however, if the standard deviation of the transmission delay measurement error sample is 3.7 × 10^−6^ s, it means that the single point detection of pseudorange measurement accuracy is 1.1 km (δ). When the SNR is greater than 40 dB, the average value of the transmission delay measurement error of the zero-crossing detection method is less than 1 × 10^−10^ s, which is equivalent to the pseudorange measurement error better than 3 cm; when the standard deviation of the transmission delay measurement error sample is 4.3 × 10^−7^ s, it means that single point detection of the pseudorange measurement accuracy is 129 m (δ).

From the simulation results, we can see that the measurement precision of the zero-crossing detection method for the Gaussian baseband waveform can theoretically reach the meter level, but the reliability of single-point measurement is not ideal.

## 5. Optimal Estimation of Timestamps

In theory, the zero-crossing detection method can make the pseudorange measurement accurate up to 10 m. However, due to the existence of channel interference, the result of a single measurement is much lower than the accuracy. In a time slot, the AIS system can transmit up to 256 bits of message, which means that the receiver will get about 200 timestamp detection results during a one frame message transmission process. This makes it possible to use the results of multi-point measurements to optimize the estimation result of transmission delay so that the pseudorange measurement accuracy is closer to the theoretical result.

### 5.1. Minimum Mean Square Error Estimation Algorithm (MMSE-EA)

A ship (considered as a user carrier of the AIS-R mode) is constantly moving, so the Doppler shift introduced by the relative motion of the ship will be included in the measurement results during one or more frames of measurement period. As the ship’s tonnage is large, its acceleration and its high order derivatives are so small that they can be neglected. Therefore, modeling the TOA measurement result in one measurement period only needs to consider the ship's uniform motion. The fitting functions are as follows

(19) $$t_{n}=a+b\times n$$

where *t_n_* is TOA of a certain bit-0-phase, and is obtained by the timestamp detection method; *n* is the bit number corresponding to the measured value *t_n_* in the bit sequence, and is obtained by matching the bit decision module and the message demodulation module; <*n*, *t_n_*> constitutes a set of measurement data pairs; *a* is the reception time of the first bit-0-phase in the frame message; *b* is the bit period of the digital message, and is affected by the Doppler shift caused by ship motion and the receiver clock frequency offset.

Based on the obtained series of measurement data pairs, the parameters of the fitting function are estimated optimally. The optimal criterion is the minimum mean square error, and the estimated result is

(20) $$\{\begin{array}{l} a=\frac{\left(\sum_{i=1}^{m}t_{n,i}\right)\left(\sum_{i=1}^{m}n_{i}^{2}\right)-\left(\sum_{i=1}^{m}n_{i}\right)\left(\sum_{i=1}^{m}n_{i}t_{n,i}\right)}{m\left(\sum_{i=1}^{m}n_{i}^{2}\right)-{\left(\sum_{i=1}^{m}n_{i}\right)}^{2}}\\ b=\frac{m\left(\sum_{i=1}^{m}n_{i}t_{n,i}\right)-\left(\sum_{i=1}^{m}n_{i}\right)\left(\sum_{i=1}^{m}t_{n,i}\right)}{m\left(\sum_{i=1}^{m}n_{i}^{2}\right)-{\left(\sum_{i=1}^{m}n_{i}\right)}^{2}} \end{array}$$

where *m* is the number of measurement data pairs; <*n_i_*, *t_n,i_*> are the i-th measurement data pairs, where *n_i_* is the corresponding bit number in the AIS message frame for the set of measurement data pairs; and *t_n,i_* is the receiving time of the corresponding timestamp.

The least square method is a statistical method that takes the least mean square error as the best estimation standard [27,28,29]. Generally, the larger the number of samples, the more accurate the estimation result. In order to evaluate the pseudorange measurement accuracy of the algorithm, Monte Carlo simulation was conducted on the estimation results with different packet lengths.

**Figure 9:**
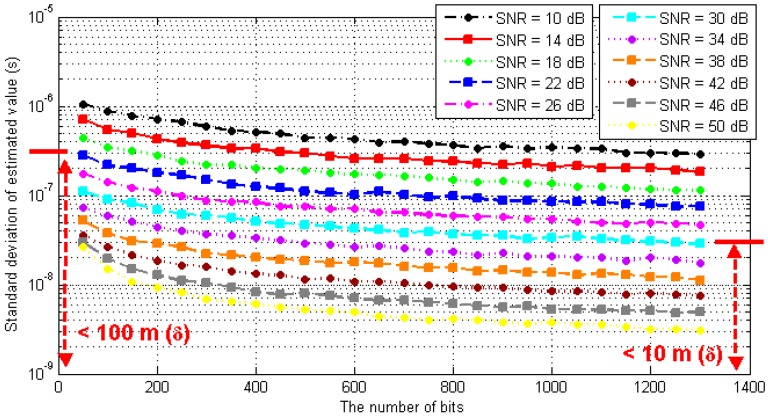
Performance analysis of optimal estimation algorithm.

Under the conditions of different SNRs and the numbers of packets, the simulation test was carried out by using the timestamp detection method and the optimal estimation algorithm proposed in this paper. The simulation was repeated 1000 times under each condition, and the standard deviation of the measured results was calculated. The simulation results are shown in Figure 9.

As seen in the above figure, the longer the packet length, the smaller the SNR, and the higher the accuracy of the pseudorange measurement obtained from the optimal estimation. Under the condition that the packet length is more than 1250 bits; when the SNR is better than 10 dB, the standard deviation of TOA estimation is better than 2.97 × 10^−7^ s, which means that the corresponding pseudorange measurement error is better than 100 m (δ). When the SNR is better than 30 dB, the standard deviation of TOA estimation is better than 2.95 × 10^−8^ s, which means the corresponding pseudorange measurement error is better than 10 m (δ).

### 5.2. Optimization Algorithm Based on α Difference Filter (OA-αDF)

According to the above discussion, we can see that for the optimal estimation algorithm, when the number of samples is larger, the precision of the pseudorange estimation is higher. However, due to the limitations of the existing AIS protocol, it is impossible to increase the message frame length indefinitely, which also limits the pseudorange estimation accuracy. By considering that the zero-crossing detection method and the differential peak detection method independently detect the same target signal, the optimal estimation results of the two detection methods can be fused by the *α* difference filtering algorithm. The essence of this algorithm is to improve the accuracy of pseudorange estimation by increasing the number of samples. The algorithm model is

(21) $$a_{df}=\left(1-\alpha \right)a_{cz}-\alpha a_{dp}$$

where *a_cz_* is the MMSE-EA result obtained by the zero-crossing detection method; *a_dp_* is the MMSE-EA result obtained by the differential peak detection method; *a_df_* is the new pseudorange estimation result after the difference filtering. *α* is the filter coefficient to be determined.

In order to determine the value of parameter *α*, the Monte Carlo numerical simulation was used for regular analysis. The simulation conditions were still as previously defined. We defined the new difference variable (*a_df_ − a_cz_*) to measure the effect of difference filtering on the accuracy of pseudorange estimation. When the variable was negative, it showed that the estimation result of the difference filtering was better than the minimum mean square error estimation based on zero-crossing detection.

**Figure 10:**
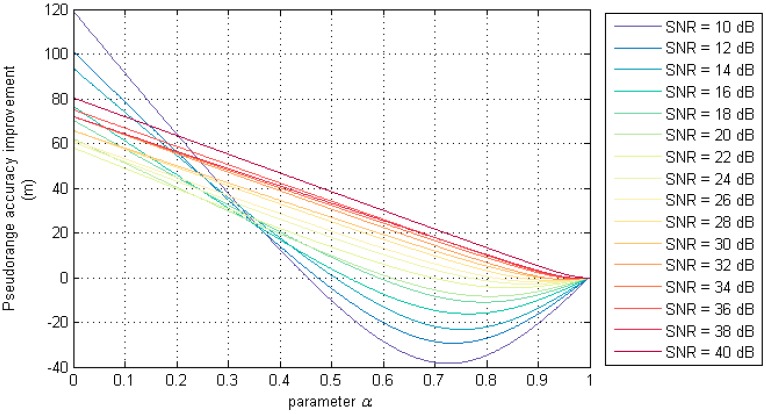
The relation between α and pseudorange accuracy under different SNR.

Figure 10 shows the improvement of pseudorange estimation accuracy under different SNR and *α* values when the AIS message frame length is 1000 bits. When *α* = 0, *a_df_* is equivalent to the differential peak detection method corresponding to the optimal estimation result *a_dp_*; when *α* = 1, *a_df_* is equivalent to the zero-crossing detection method corresponding to the optimal estimation result *a_cz_*. It can be seen from the figure that the accuracy of the pseudorange estimation results based on the differential peak detection method are inferior to the estimation method based on the zero-crossing detection method under different SNR. At the same time, it can also be seen that when the SNR is small, the difference filtering algorithm improves the pseudorange estimation results more obviously. In Figure 10, the corresponding α value of the lowest point in each curve is the value of the parameter *α* in the difference filtering algorithm under the SNR condition. 

**Figure 11:**
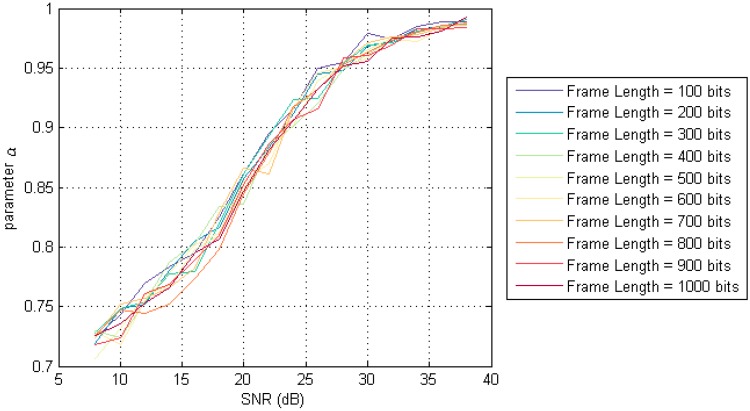
The relation between α and SNR under different frame length.

Figure 11 shows that the curves obtained under different frame lengths are substantially coincident. This indicates that the value of parameter α of the difference filtering algorithm is only related to the SNR, and has nothing to do with the length of the frame. 

Through the curve fitting method, we can get the equation of the parameter *α*, which is 

(22) α=0.1195×arctan(0.1386×SNR−2.7634)+0.85

**Figure 12:**
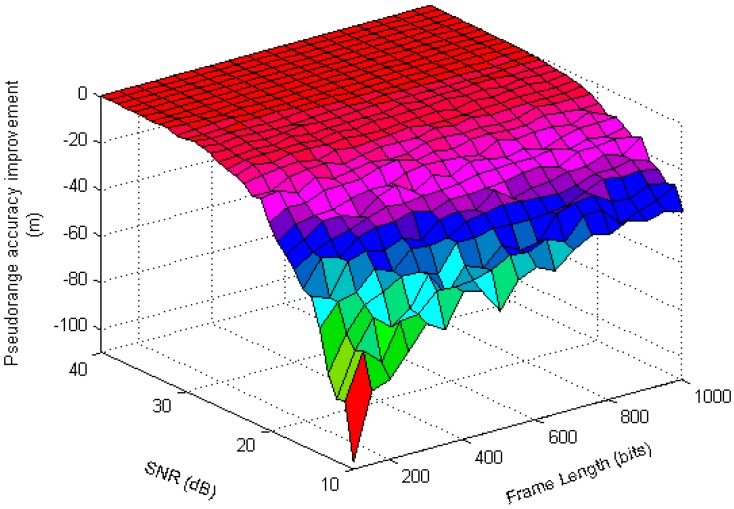
Pseudorange improvement effect of OA-α DF algorithm.

According to the difference filtering algorithm defined by Equations (21) and (22), the improvement of the pseudorange estimation accuracy under different frame length and SNR is shown in Figure 12.

As seen in Figure 12, when the SNR is less than 20 dB, the accuracy of the MMSE-EA algorithm itself is relatively poor, so when superimposed on the differential filtering, the improvement of the pseudorange accuracy is obvious. When the SNR of the baseband signal is large, the improvement effect of the OA-*α*DF algorithm to the pseudorange estimation value is not so obvious.

### 5.3. Algorithm Performance Comparison

In [23], according to the bit error rate (BER) in the AWGN channel and the characteristics of GMSK, the authors proposed a threshold of transmission delay estimation based on modified Cramer–Rao Bound. The conclusion is

(23) $$\sigma_{GMSK,bit\;edge}\geq \sqrt{MCRB_{GMSK}(\tau )}=\frac{0.12}{\sqrt{L_{0}}10^{\frac{s}{20}}}$$

where *s* is signal power in dBm; *L*_0_ is message frame length in bits; *MCRB_GMSK_*(*τ*) is the modified Cramer–Rao Bound function, *τ* represents the transmission delay; *σ_GMSK,bit edge_* is a signal transmission delay measurement with its unit in nanoseconds. 

It should be noted that the literature only gives the theoretical derivation process and conclusions based on the Cramer–Rao Bound theory, but does not give the actual measurement method, and the derivation of the document requires AIS message content as a fixed message, which greatly occupies the bandwidth of the AIS system.

**Figure 13:**
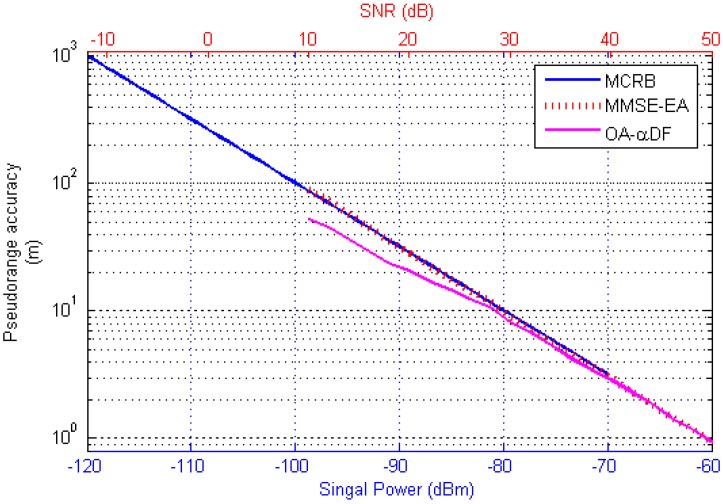
Algorithm performance comparisons.

The length of the AIS message frame at 1280 bits in the five slots mentioned in [23], the estimation result obtained by Equation (23) and the estimation result by the measurement method proposed in this paper were compared. The corresponding conclusion is shown in Figure 13.

The transmission delay threshold given in [23] was concerned with the signal power, and the measurement method and optimal estimation algorithms presented here focus on the SNR of the baseband signal. Both are descriptions of received signal power. It can be seen in Figure 13, that the performance of the MMSE-EA algorithm (based on the zero-crossing detection) is in agreement with the theoretical threshold characteristic given by Equation (23). The OA-*α*DF algorithm proposed in this paper can effectively improve the accuracy of pseudorange estimation under low SNR conditions. 

Through the comparison of the MCRB and MMSE-EA curves in Figure 13, it was determined that the noise intensity of the baseband signal corresponding to the MCRB curve was −107 dBm, while the channel noise given in [23] was −117 dBm, with a 10 dB difference between them. This is because [23] focused on radio frequency (RF) signal noise, and this paper is concerned with baseband signal noise, so in the actual measurement circuit, the noise filter performance of the RF circuit and demodulation module will affect final measurement accuracy. 

## 6. Field Test Verification

**Figure 14:**
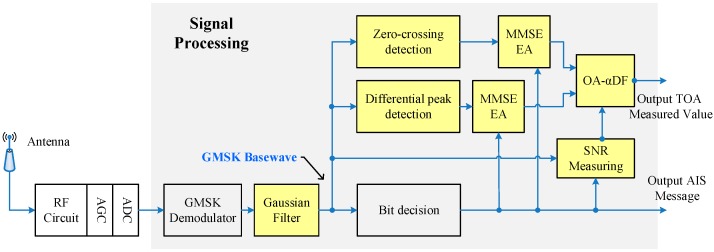
AIS transmission delay measurement scheme block diagram.

Based on the above results, we present a pseudorange measurement scheme based on the existing AIS communication system, and the specific block diagram is shown in Figure 14:

The RF circuit processes the received wireless signal by the frequency shift to obtain the intermediate frequency (IF) signal with a central frequency of 455 KHz. When the IF signal is processed by the automatic gain control module, the received signals of different intensities are normalized. The digital IF signal (obtained by sampling the analog IF signal through an analog-to-digital converter (ADC)) is sent to the signal processing module, which is implemented by FPGA for data decoding and TOA measurements.

**Figure 15:**
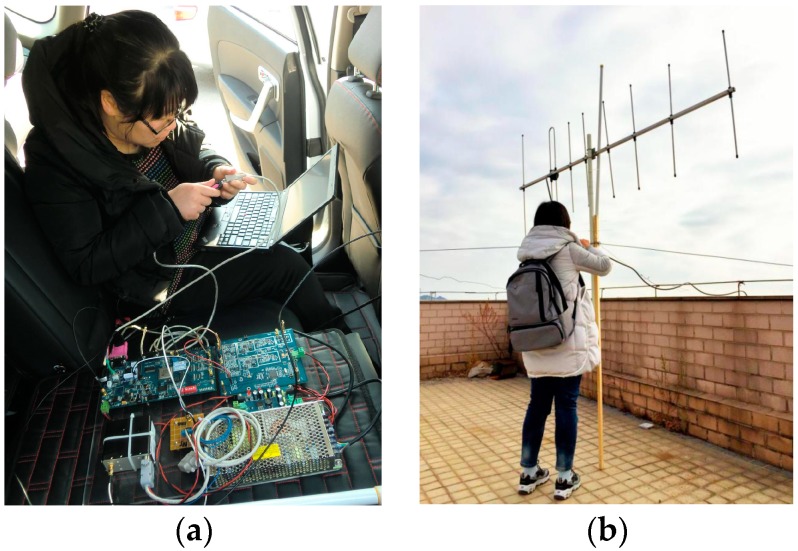
Science of field test. (**a**) Engineering prototype; and (**b**) Yagi–Uda antenna.

In the signal processing module, the Gaussian baseband waveform (the research object in this paper) is recovered by the GMSK demodulation module from the digital IF signal. We used the coherent demodulation method based on phase locked loop [30], which can theoretically improve the SNR by 3 dB. The subsequent signal processing flow is the engineering realization of the pseudorange measurement scheme described above. The bit numbers corresponding to the timestamps were provided by the message sequence recovered by the bit decision module. When a frame of message demodulation was completed, the signal processing module could output a TOA measurement value. The engineering prototype is shown in Figure 15a. 

In order to remove the error caused by the clock source interference at both ends of the transmitter and receiver, we used a rubidium atomic clock which had been synchronized by GNSS as the clock source. Additionally, we simultaneously used a Yagi–Uda antenna (Figure 15b) as the transmitting and receiving antenna to reduce the influence of multipath interference on the measurement results [31,32]. 

**Figure 16:**
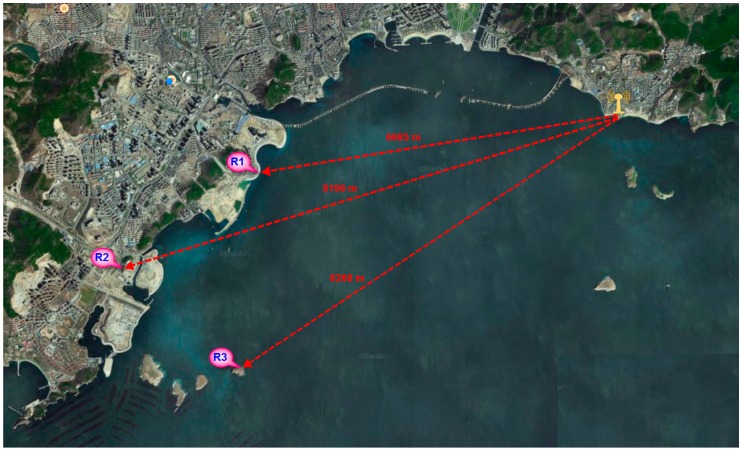
Schematic diagram of field test site layout.

The field test site was located in Xinghai Bay, Dalian City, Liaoning Province. To ensure that there was no obstruction between the two points, we set up one fixed transmitting station and three receiving stations (Figure 16). The distance shown in the figure is the actual distance calculated from the longitude and latitude coordinates. The accuracy of the pseudorange measurement was statistically analyzed by the method of fixed-point multiple tests.

**Figure 17:**
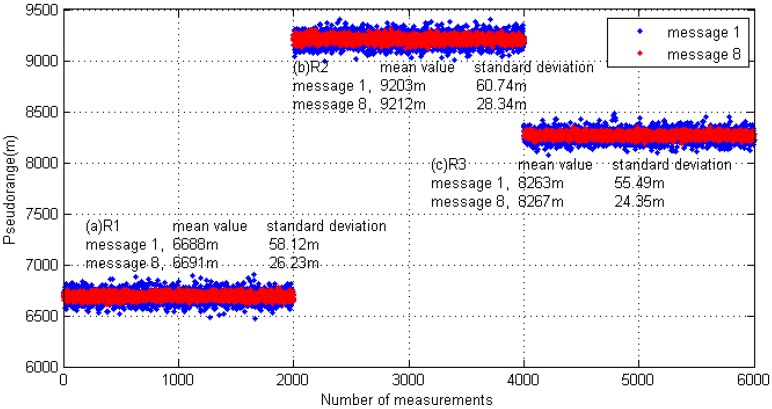
Field measurement data analysis results.

The test was divided into short- and long-message ranging. In the short-message ranging experiment, the transmitter station only sent Message 1 and the length of the link layer packet was approximately 224 bits. In the long message ranging experiment, the transmitting station only sent Message 8, and the length of the link layer packet bit was approximately 1064 bits. In both test conditions, the contents of the transmitted message were not fixed, but encapsulated according to real-time information. In order to protect the transmitter's power amplifier, the transmission interval was set to five seconds. For different test content, each receiving point continuously collected about three hours of test data. The pseudorange measurement results are shown in Figure 17.

Figure 17 shows that the pseudorange measurement scheme proposed in this paper is valid under different distance conditions. For the test prototype, in the case of a one-slot short message, the pseudorange measurement accuracy was better than 60 m (δ); and in the case of a five-slot long message, the pseudorange measurement accuracy was better than 28 m (δ).

## 7. Conclusions

This paper studied the most basic pseudorange measurement problem in AIS-R mode in depth. The theoretical analysis and simulation verification of the key problems in the AIS signal transmission delay measurement (such as timestamp option, the detection method of the timestamp object, and the optimization of the measurement accuracy) are carried out. On this basis, a set of completed measurement programs is put forward. The results of this study make the AIS system add a new pseudorange measurements function without changing the original equipment.

By considering that the AIS uses the frequency modulation method, the carrier phase was not suitable as a timestamp to be measured. Instead, the bit 0 phase was selected as the timestamp object. Through the mathematical modeling analysis for an AIS message frame (in addition to each two bits of the header and the tail), the timestamp objects corresponding to the other bits have two characteristics: the amplitude is equal to 0 and the differential result is the peak. The comparison shows that in the AWGN channel, the accuracy of the zero-crossing detection method was higher than that of the differential peak detection method. As the ship is a low-speed and low-dynamic motion carrier, the measurement accuracy of the pseudorange is greatly improved by the optimal estimation algorithm based on the MMSE. The Monte Carlo simulation results show that the pseudorange measurement accuracy of the optimal estimation algorithm based on the zero-crossing detection method was consistent with the conclusion deduced from the modified Cramer–Rao Bound in [23]. Furthermore, the measurement results obtained by combining the zero-crossing detection and differential peak detection were further improved under low SNR conditions. 

The final field test results show that the pseudorange measurement accuracy of the functional prototype was better than 28 m (σ). For such large vessels as ships, the results have been able to meet the positioning requirements. Furthermore, as the measurement scheme proposed in this paper had no special requirements for the message format, the channel capacity of the AIS was not increased. In conclusion, the theoretical measurement accuracy has achieved or exceeded that of other visible studies. 

Our future work will look at optimizing the RF circuit by reducing system noise and optimizing the performance of the pre-filter to improve the pseudorange measurement accuracy of the principle prototype.

## Figures and Tables

**Table 1:** Relationship between bit-0-phase amplitude error and bit number in AIS packet. ^1^

Bit Number	1	2	......	55	56
Bit-0-Phase amplitude error	1.268 × 10^−3^	7.826 × 10^−10^	0	7.826 × 10^−10^	−1.268 × 10^−3^

^1^ The package length *N* = 56.
